# Supplementary material for: Influence of Carbon Black and Silica Fillers with Different Concentrations on Dielectric Relaxation in Nitrile Butadiene Rubber Investigated by Impedance Spectroscopy
Source: Polymers (Basel). 2021 Dec 31;14(1):155. doi: 10.3390/polym14010155 (PMC8747209; doi:10.3390/polym14010155)
Supplement: Supplementary file 1 [file polymers-14-00155-s001.zip › polymers-1507601-supplementary.pdf]

Supplementary Materials

Figure S1 represents the simulation example for the measured complex permittivity in NBR-H20. On the left side of Figure S1, the real and imaginary parts of the permittivity are shown in a three-dimensional graph as a function of both temperature (233 K – 403 K) and frequency (0.01 Hz - 1 MHz). The value on x ,y and z axis indicates frequency, temperature and permittivity, repectivly. The red solid line indicates the real part of the permittivity on z axis, and the blue line corresponds to the imaginary part. To precisely extract the complex permittivity from the measured capacitance and loss tangent data, the area and thickness for the specimen shown below the three-dimensional graph was entered when the data were imported.

The right side of Figure S1 shows the corresponding two-dimensional permittivity spectra versus frequency in NBR-H20 at 298 K, deconvoluted by two HN models and one conductivity term in equation (6) using a modified dispersion analyzer program [1, 2]. The red and blue cross symbols (x) indicate the real and imaginary parts of the measured permittivity, respectively. The red and blue solid lines show the simulated results obtained by superposing the two HN and conductivity models. The simulation was based upon the least square method that reduced the difference between the measured data and fitting results. In the model information, the parameters σ, N, and ε_0 for the global model shown in the first term of equation (6) and the parameters (α, β, f_0, and $\varepsilon_0 - \varepsilon_\infty$) for the HN model in the third term of equation (6) are obtained. The parameters for the selected model functions are displayed in an unknown parameter list. The min and max values indicate a limited range of simulation, and their values correspond to the results obtained from the simulation at the corresponding temperature.

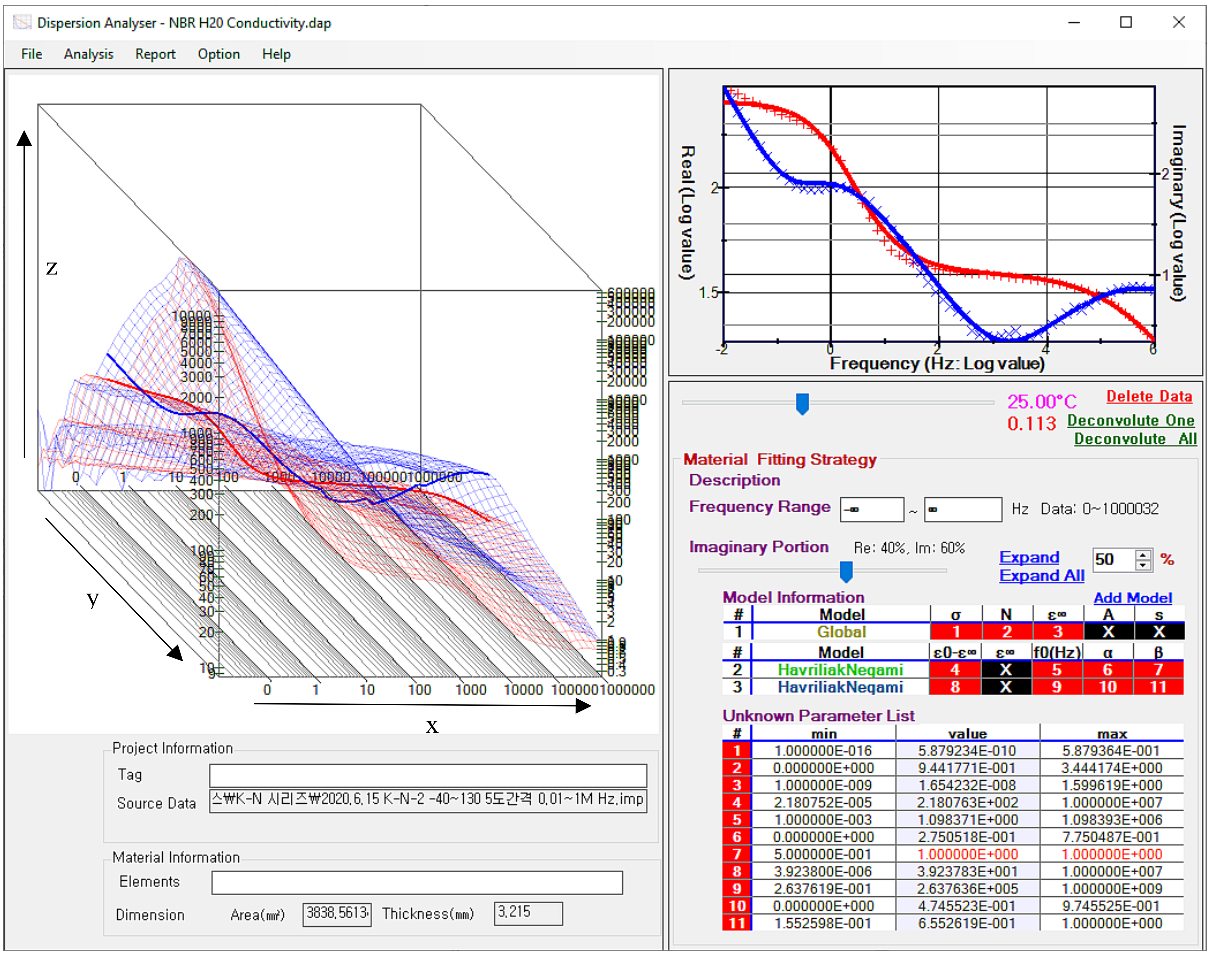

Figure S1. Simulation result for the measured complex permittivity in NBR-H20 at 298 K using the modified dispersion analysis program.

Table S1. ASTM classification for type of carbon blacks.

Name	Standard abbreviation	ASTM designation	Particle size(nm)	Tensile strength(Mpa)	Relative laboratory abrasion	Relative roadwear abrasion
Super Abrasion Furnace	SAF	N110	20-25	25.2	1.35	1.25
Intermediate SAF	ISAF	N220	24-33	23.1	1.25	1.15
High Abrasion Furnace	HAF	N330	28-36	22.4	1.00	1.00
Easy Processing Channel	EPC	N300	30-35	21.7	0.80	0.90
Fast Extruding Furnace	FEF	N550	39-55	18.2	0.64	0.72
High Modules Furnace	HMF	N660	49-73	16.1	0.56	0.66
Semi-Reinforcing Furnace	SRF	N770	70-96	14.7	0.48	0.60
Fine Thermal	FT	N880	180-200	12.6	0.22	-
Medium Thermal	MT	N990	250-350	9.8	0.18	-

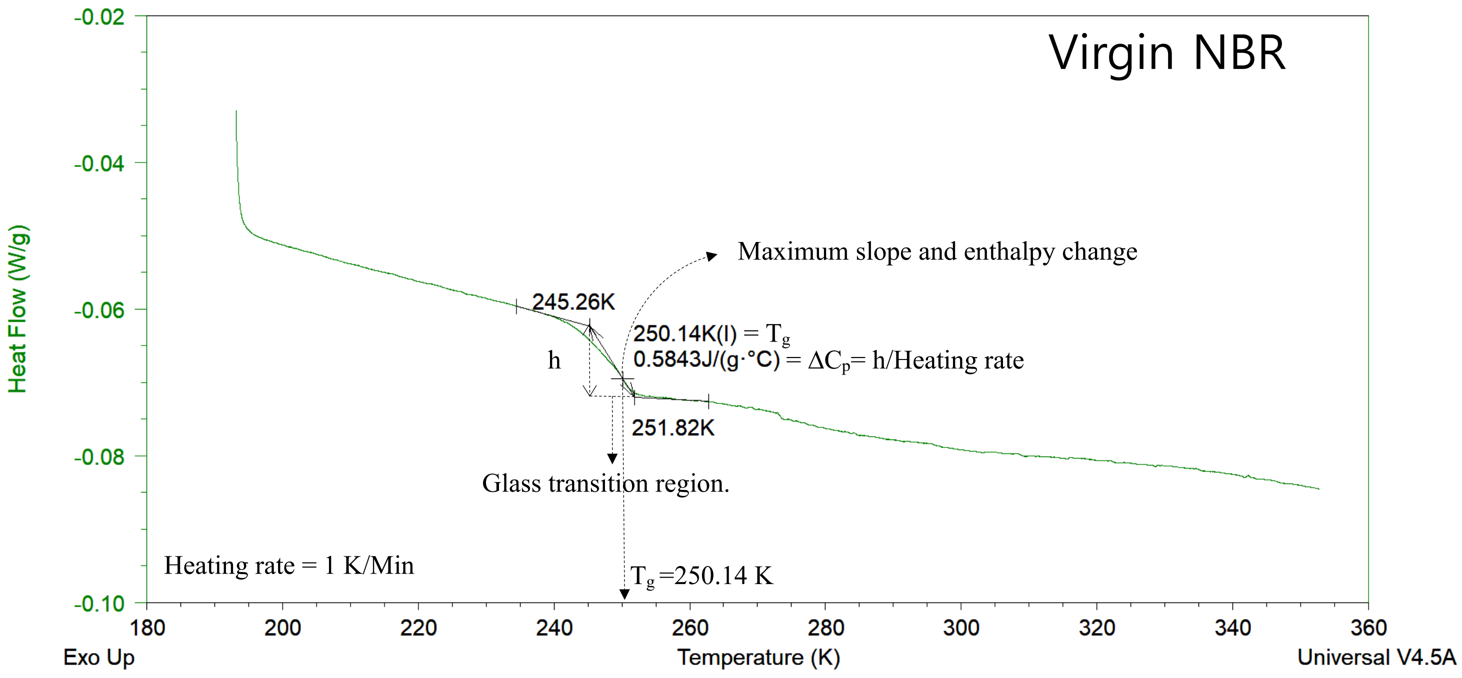

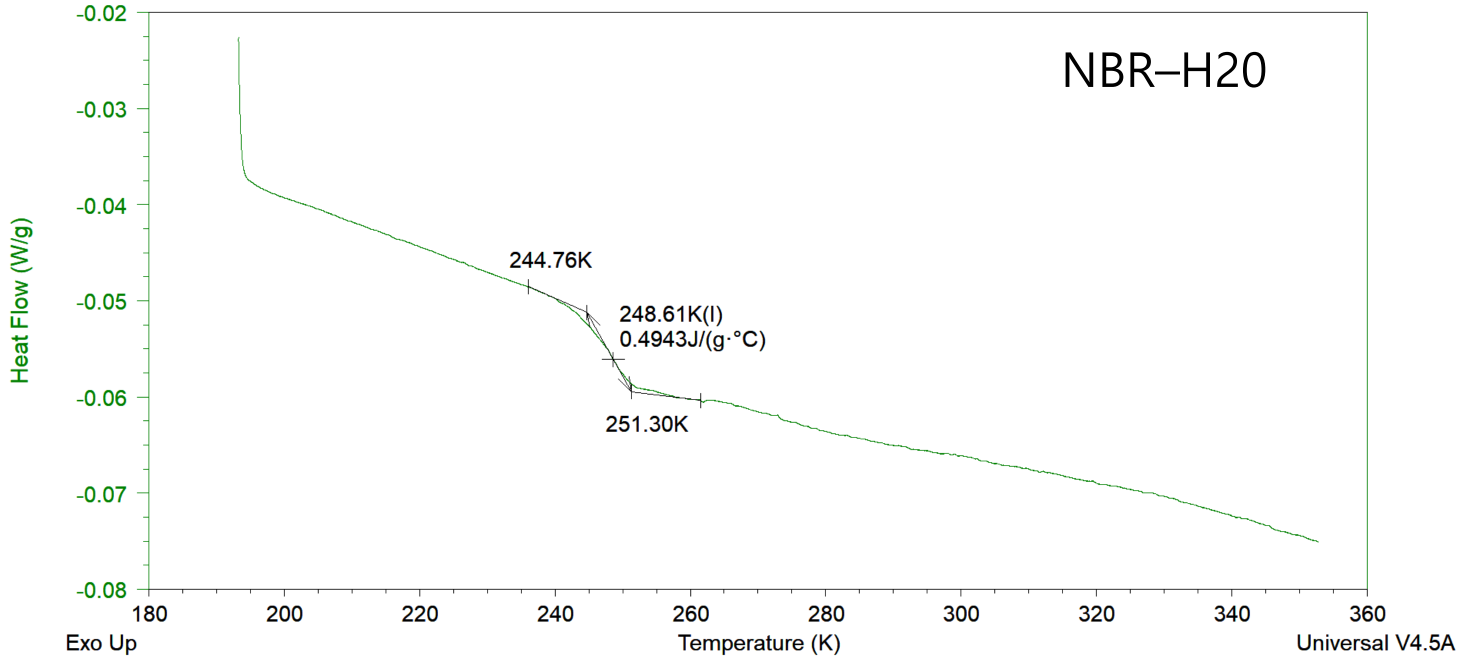
NBR–H20
-0.02
-0.03
-0.04
-0.05
-0.06
-0.07
-0.08
Heat Flow (W/g)
244.76K
248.61K(I)
0.4943J/(g·°C)
251.30K
180
200
220
240
260
280
300
320
340
360
Exo Up
Temperature (K)
Universal V4.5A

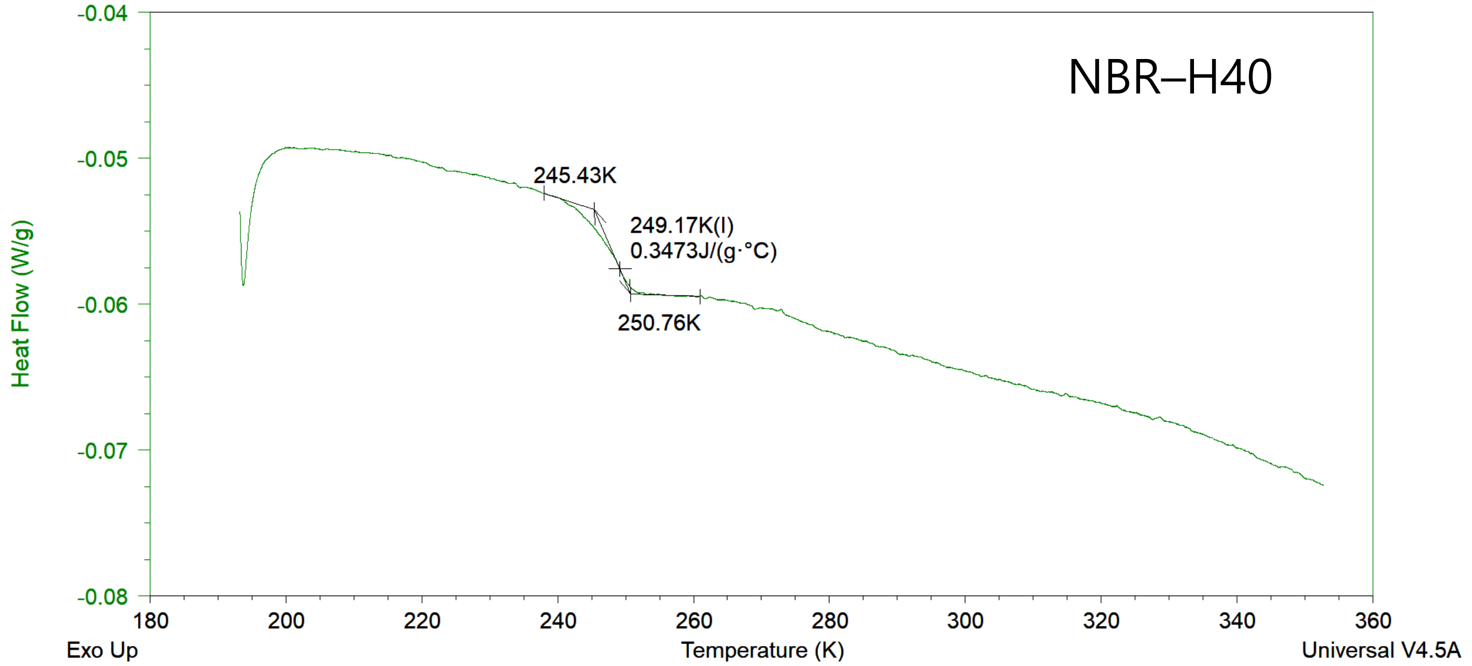
NBR–H40
-0.04
-0.05
-0.06
-0.07
-0.08
Heat Flow (W/g)
245.43K
249.17K(I)
0.3473J/(g·°C)
250.76K
180
200
220
240
260
280
300
320
340
360
Exo Up
Temperature (K)
Universal V4.5A

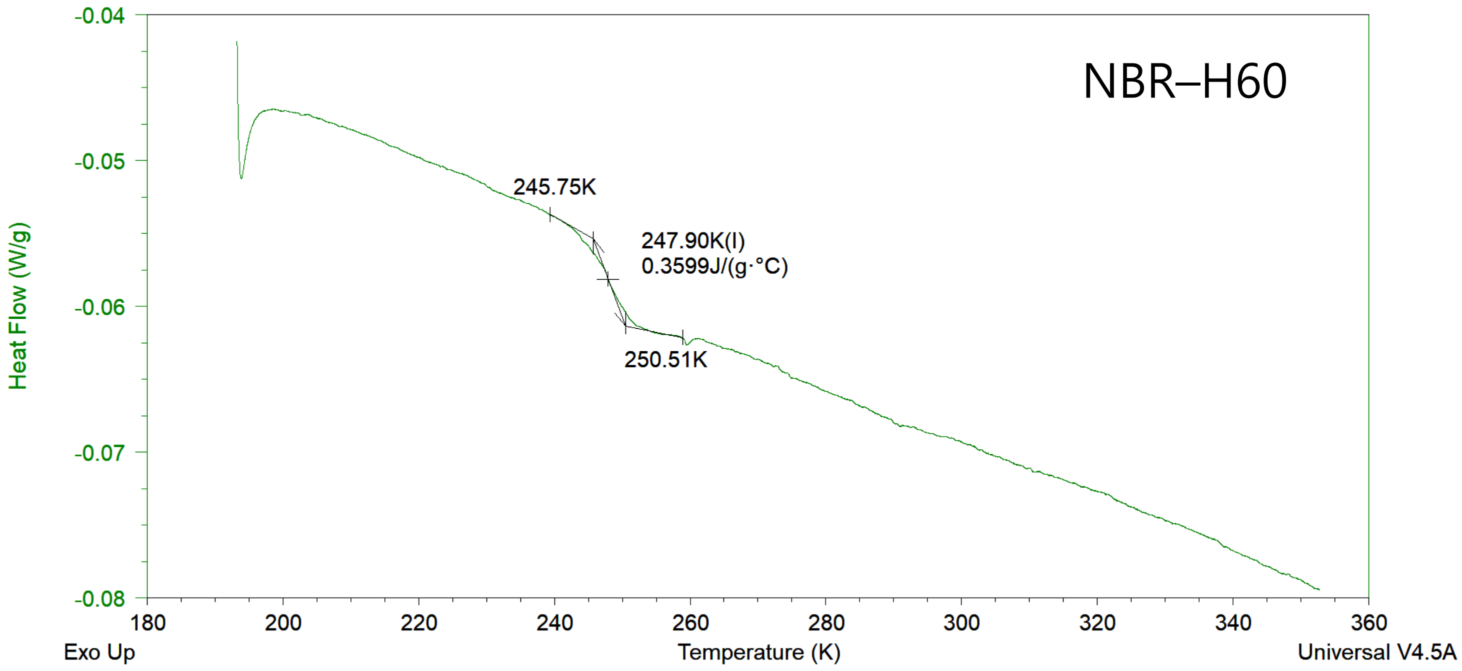
NBR–H60
245.75K
247.90K(I)
0.3599J/(g·°C)
250.51K
Heat Flow (W/g)
-0.04
-0.05
-0.06
-0.07
-0.08
180
200
220
240
260
280
300
320
340
360
Exo Up
Temperature (K)
Universal V4.5A

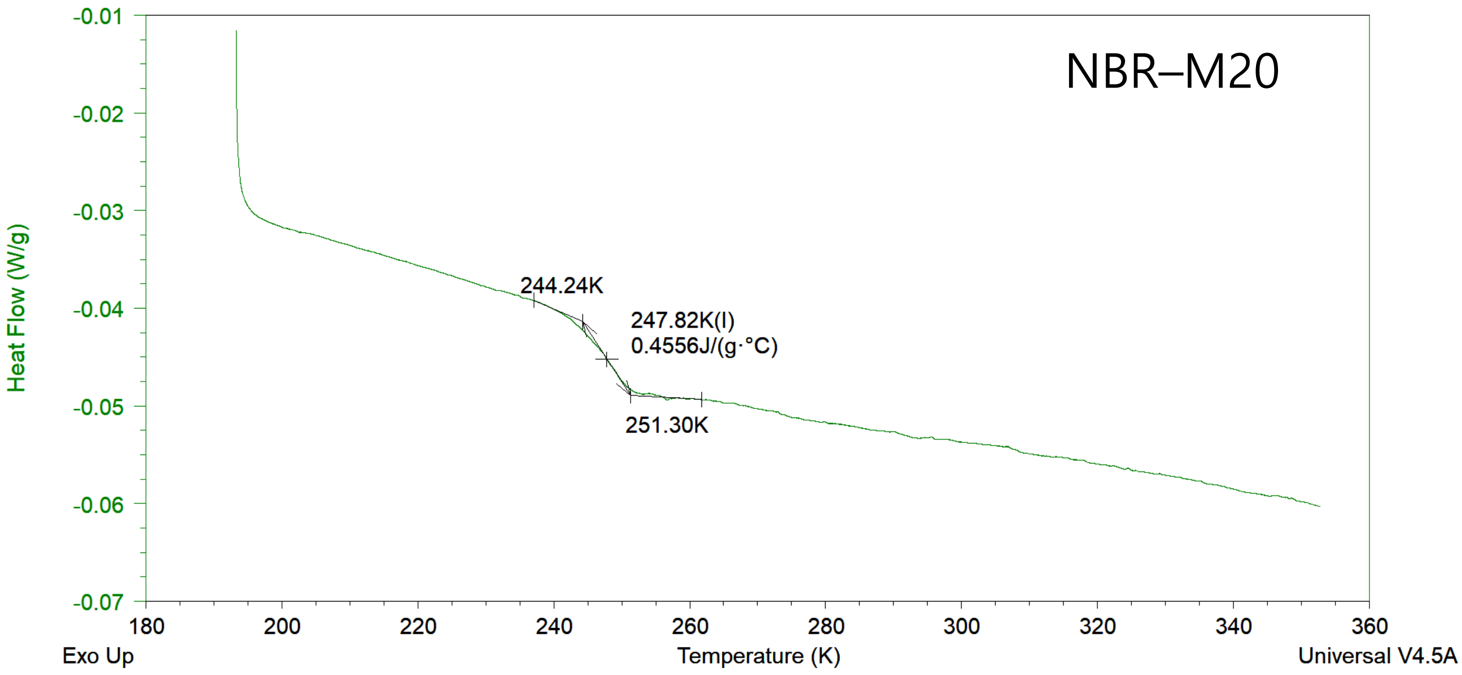
NBR–M20
244.24K
247.82K(I)
0.4556J/(g·°C)
251.30K
Heat Flow (W/g)
-0.01
-0.02
-0.03
-0.04
-0.05
-0.06
-0.07
180
200
220
240
260
280
300
320
340
360
Exo Up
Temperature (K)
Universal V4.5A

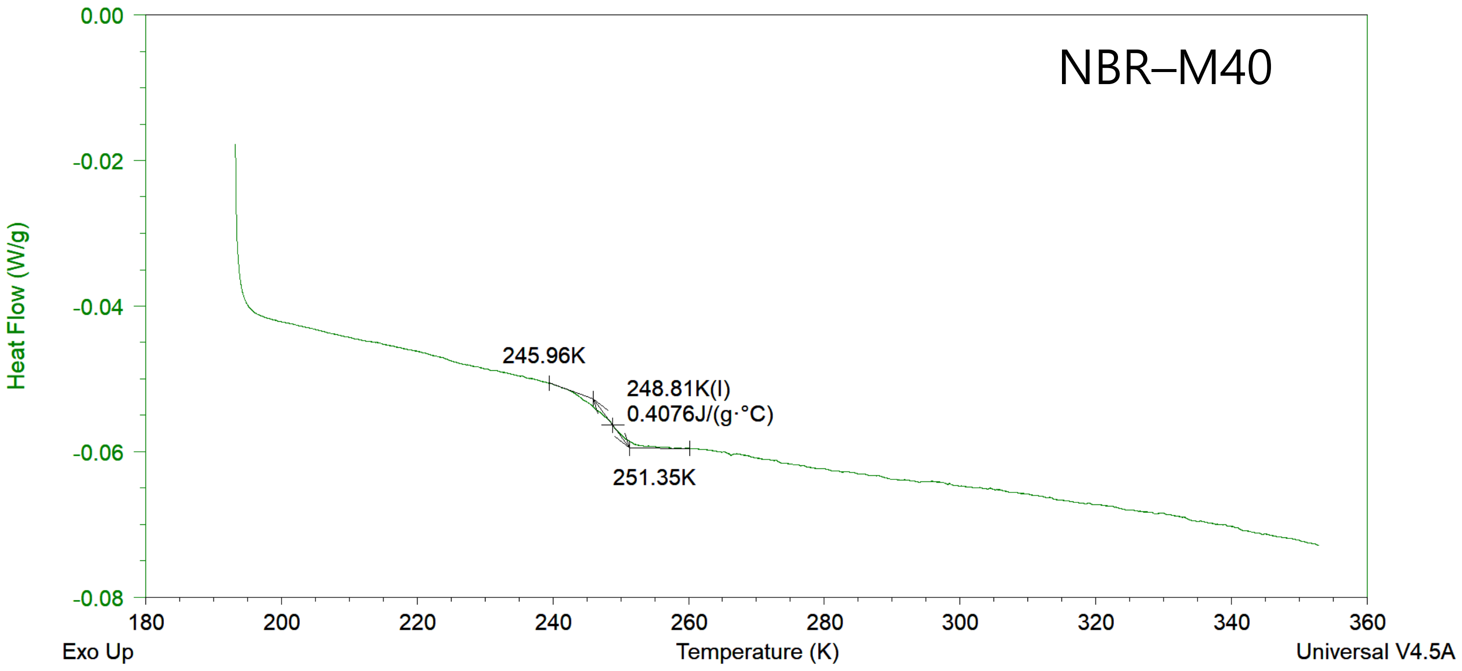
NBR–M40
Heat Flow (W/g)
0.00
-0.02
-0.04
-0.06
-0.08
245.96K
248.81K(I)
0.4076J/(g·°C)
251.35K
180
200
220
240
260
280
300
320
340
360
Exo Up
Temperature (K)
Universal V4.5A

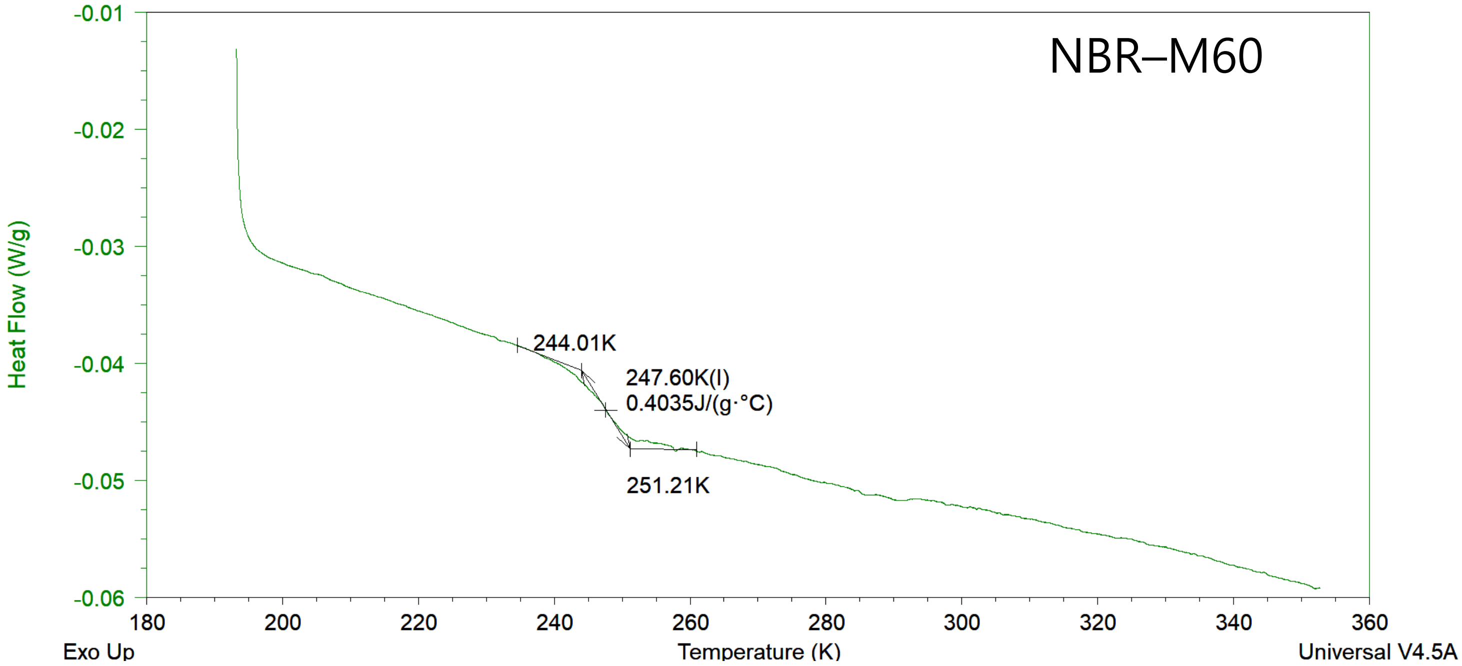
NBR–M60
Heat Flow (W/g)
-0.01
-0.02
-0.03
-0.04
-0.05
-0.06
244.01K
247.60K(I)
0.4035J/(g·°C)
251.21K
180
200
220
240
260
280
300
320
340
360
Exo Up
Temperature (K)
Universal V4.5A

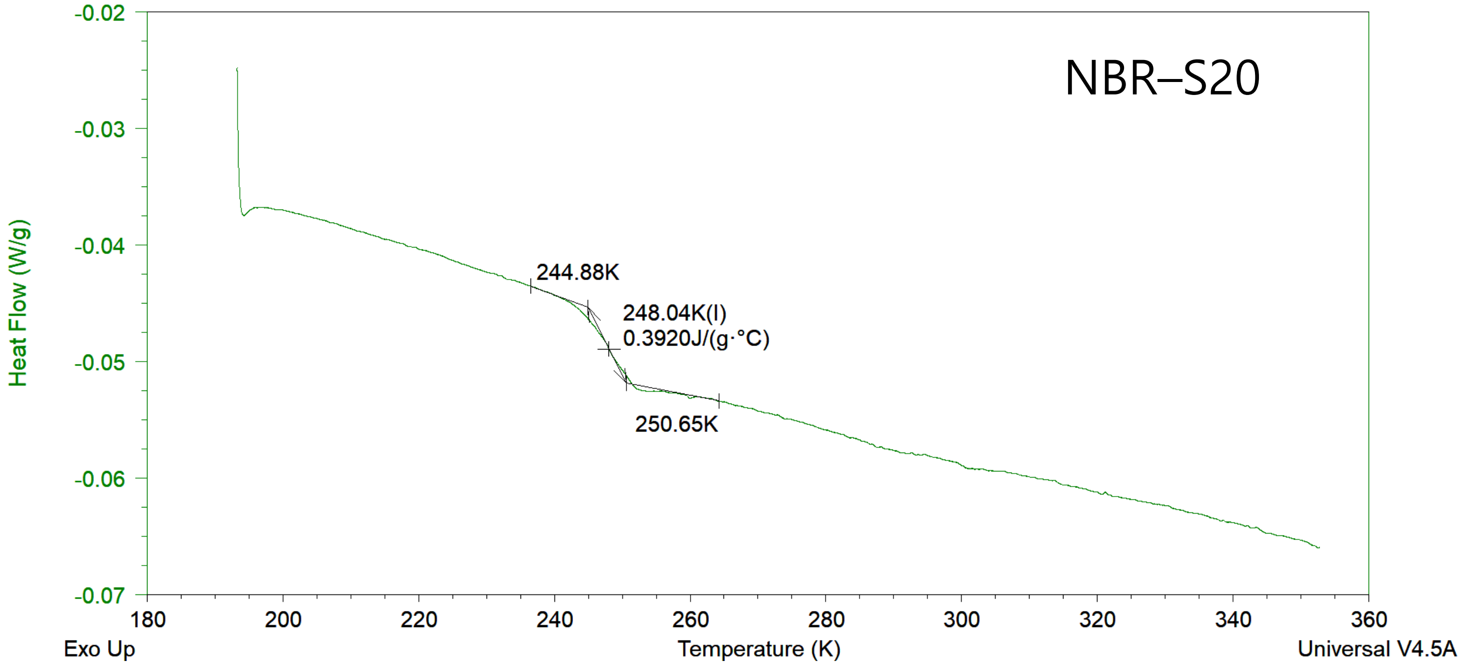
NBR–S20
Heat Flow (W/g)
-0.02
-0.03
-0.04
-0.05
-0.06
-0.07
244.88K
248.04K(I)
0.3920J/(g·°C)
250.65K
180
200
220
240
260
280
300
320
340
360
Exo Up
Temperature (K)
Universal V4.5A

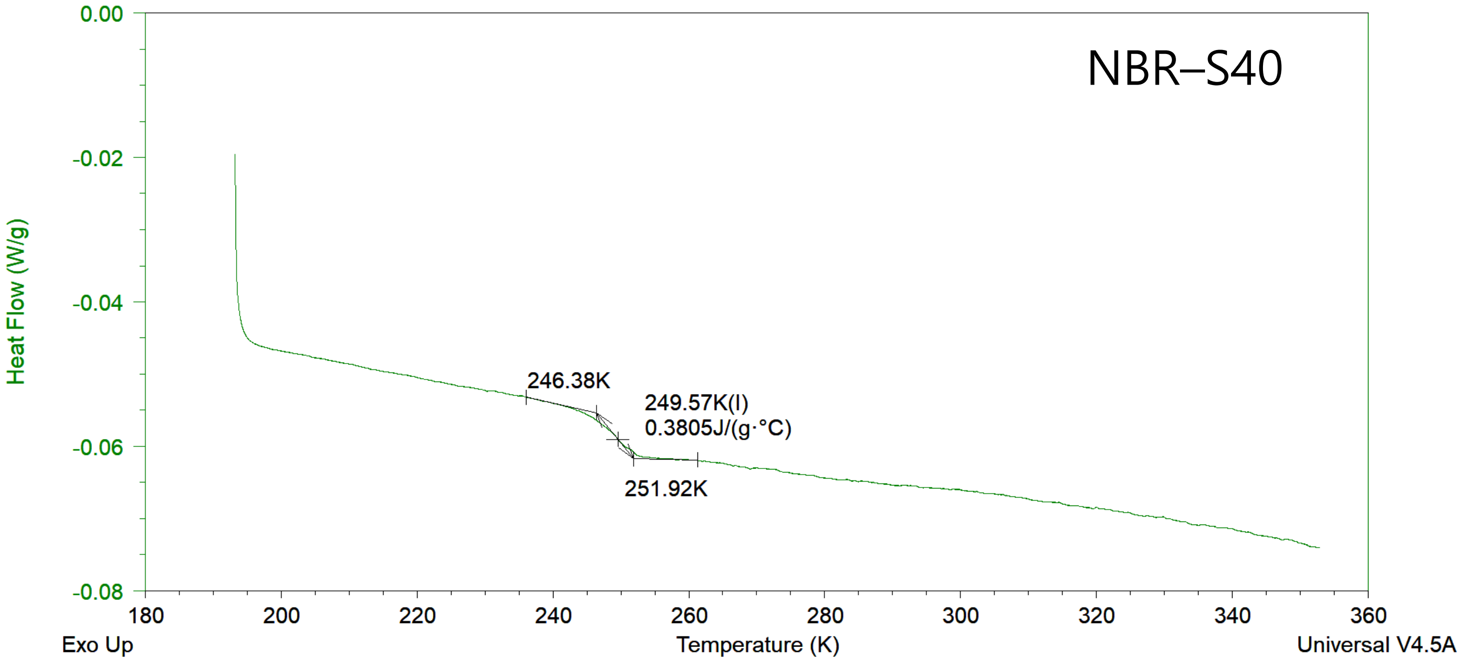
NBR–S40
Heat Flow (W/g)
0.00
-0.02
-0.04
-0.06
-0.08
246.38K
249.57K(I)
0.3805J/(g·°C)
251.92K
180
200
220
240
260
280
300
320
340
360
Exo Up
Temperature (K)
Universal V4.5A

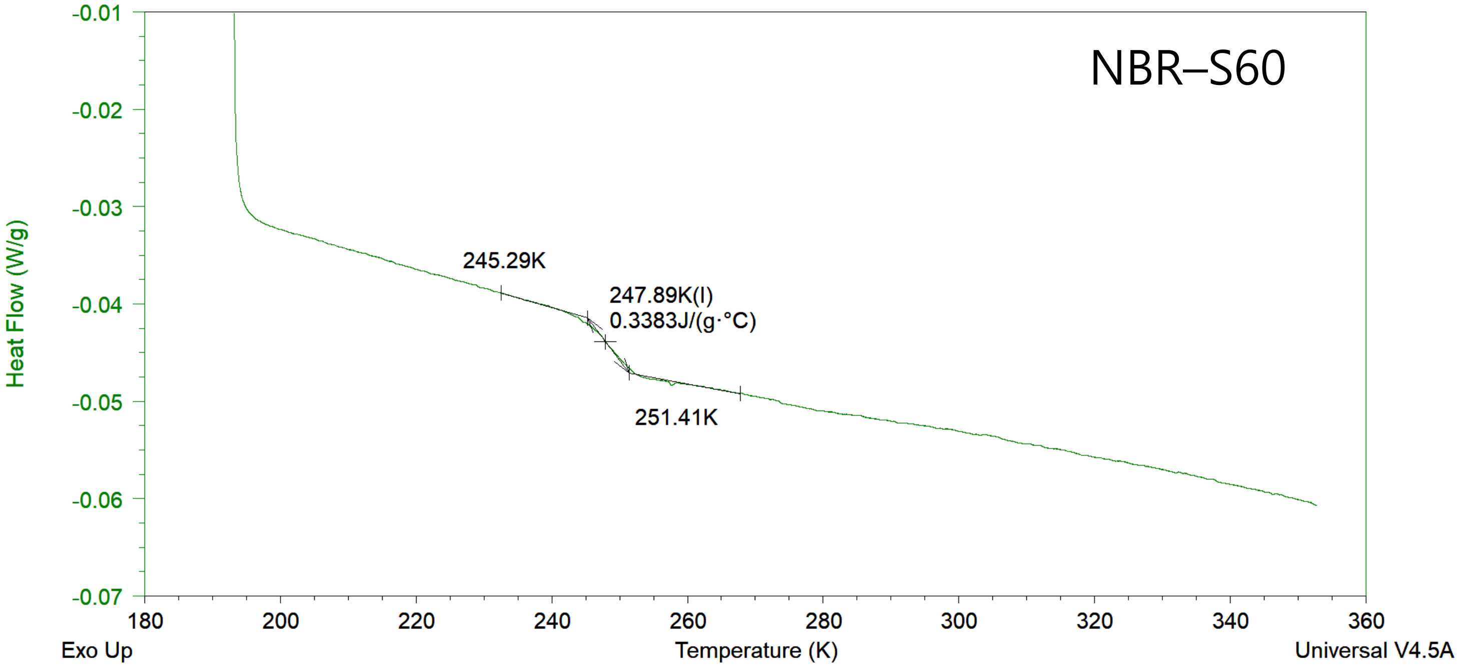

Figure S2. The measured heat flow curves of our NBR specimens by using DSC instrument. In virgin NBR, it shows the process of obtaining glass transition temperature and ΔC_p. The glass transition is the temperature at the maximum slope(maximum enthalpy) in glass transition region.

References

1. J.K. Jung, Y.I. Moon, G.H. Kim, N.H. Tak, Characterization of dielectric relaxation process by impedance spectroscopy for polymers: nitrile butadiene rubber and ethylene propylene diene monomer, J. Spectrosc. (2020) 8815492.

2. Y.I. Moon, J.K. Jung, G.H. Kim, K.S. Chung, Observation of the relaxation process in fluoroelastomers by dielectric relaxation spectroscopy, Phys. B Condens. Matter 608 (2021) 412870.
